# Tools for the Individualized Therapy of Teicoplanin for Neonates and Children

**DOI:** 10.1128/AAC.00707-17

**Published:** 2017-09-22

**Authors:** V. Ramos-Martín, M. N. Neely, K. Padmore, M. Peak, M. W. Beresford, M. A. Turner, S. Paulus, J. López-Herce, W. W. Hope

**Affiliations:** a Antimicrobial Pharmacodynamics and Therapeutics, Institute of Translational Medicine, University of Liverpool, Liverpool, United Kingdom; b Alder Hey Children's NHS Foundation Trust, Liverpool, United Kingdom; c Laboratory of Applied Pharmacokinetics and Bioinformatics, The Saban Research Institute and Division of Pediatric Infectious Diseases, Children's Hospital Los Angeles, University of Southern California, Los Angeles, California; d Liverpool Women's NHS Foundation Trust, Liverpool, United Kingdom; e Department of Women's and Children's Health, Institute of Translational Medicine, University of Liverpool, Liverpool, United Kingdom; f Servicio de Cuidados Intensivos Pediátricos, Hospital General Universitario Gregorio Marañón, Madrid, Spain; g Instituto de Investigación Sanitaria del Hospital Gregorio Marañón, Facultad de Medicina, Universidad Complutense de Madrid, Madrid, Spain

**Keywords:** children, individualized therapy, population pharmacokinetics, teicoplanin

## Abstract

The aim of this study was to develop a population pharmacokinetic (PK) model for teicoplanin across childhood age ranges to be used as Bayesian prior information in the software constructed for individualized therapy. We developed a nonparametric population model fitted to PK data from neonates, infants, and older children. We then implemented this model in the BestDose multiple-model Bayesian adaptive control algorithm to show its clinical utility. It was used to predict the dosages required to achieve optimal teicoplanin predose targets (15 mg/liter) from day 3 of therapy. We performed individual simulations for an infant and a child from the original population, who provided early first dosing interval concentration-time data. An allometric model that used weight as a measure of size and that also incorporated renal function using the estimated glomerular filtration rate (eGFR), or the ratio of postnatal age (PNA) to serum creatinine concentration (SCr) for infants <3 months old, best described the data. The median population PK parameters were as follows: elimination rate constant (Ke) = 0.03 · (wt/70)^−0.25^ · Renal (h^−1^); *V* = 19.5 · (wt/70) (liters); Renal = eGFR^0.07^ (ml/min/1.73 m^2^), or Renal = PNA/SCr (μmol/liter). Increased teicoplanin dosages and alternative administration techniques (extended infusions and fractionated multiple dosing) were required in order to achieve the targets safely by day 3 in simulated cases. The software was able to predict individual measured concentrations and the dosages and administration techniques required to achieve the desired target concentrations early in therapy. Prospective evaluation is now needed in order to ensure that this individualized teicoplanin therapy approach is applicable in the clinical setting. (This study has been registered in the European Union Clinical Trials Register under EudraCT no. 2012-005738-12.)

## INTRODUCTION

The pharmacokinetics (PK) of teicoplanin are highly variable in children and neonates (1, 2). Weight-based dosing is advocated (3). Weight affects estimates of clearance and has been incorporated into structural PK models using linear and allometric scaling functions (2, 4–7). Different levels of renal function (quantified in terms of the serum creatinine concentration (SCr) and estimated glomerular filtration rate [eGFR]) also explain a portion of PK variability in adults and children (7, 8). However, most of the interpatient variability in PK remains unexplained (7, 9). Consequently, nomograms based on simple covariates cannot be used to adjust dosages to achieve therapeutic targets that are safe and maximally effective.

The ratio of the area under the concentration-time curve (AUC) to the MIC is the pharmacodynamic index that best links teicoplanin drug exposure with the observed effect against methicillin-resistant Staphylococcus aureus (MRSA) (38). Nevertheless, the measure of drug exposure most widely used to guide therapeutic drug monitoring (TDM) is the predose concentration (*C*_min_ [measured in milligrams per liter] or trough concentration) (10). A *C*_min_ target of ≥15 mg/liter by days 3 to 5 of therapy is recommended for most clinical indications (11). This target has recently been increased in the eMC's Summary of Product Characteristics (SPC) to 20 mg/liter for the treatment of deep-seated infections (bone and joint infections) and 30 to 40 mg/liter for infective endocarditis (11). Currently recommended targets are based on small retrospective studies in adults that have explored the relationship between teicoplanin drug exposure and clinical outcomes (12, 13). More recently, daily AUCs of >750 to 800 mg · h/liter by day 3 of therapy have been linked to microbiological cure of adults with MRSA infections (14, 15).

In this study, we report the development of a nonparametric population PK model of teicoplanin in hospitalized neonates, infants, and older children (up to the age of 16 years). We then describe the application of this model for the construction of software that provides support for individualizing the dose of teicoplanin. Such an approach enables the achievement of desired drug exposure targets in an optimally precise manner and at any time during the therapeutic course. This approach constitutes a further extension of our broad goal of developing the tools and knowledge to deliver optimized antimicrobial therapy for neonates and children.

## RESULTS

### Demographics.

The demographics and clinical characteristics of the 57 patients used in the population PK model are summarized in Table 1. The total population (*n* = 57) was comprised of neonates (*n* = 18), infants and toddlers 1 to 23 months old (*n* = 16), children 2 to 11 years old (*n* = 20), and children 11 to 16 years old (*n* = 3). The majority of patients (*n* = 23 [40.35%]) were recruited from the intensive-care unit (ICU), in most cases after cardiac surgery. Other subjects included oncologic patients with febrile neutropenia (*n* = 17 [29.8%]) and patients with general medical (*n* = 8 [28.1%]) and cardiac (*n* = 1 [1.75%]) conditions. A total of 394 PK samples were available for analysis; each patient contributed a mean of 5.3 observations (neonates) or 7.6 observations (older children).

**TABLE 1:** Demographics and clinical characteristics of patients

Demographic or clinical characteristic^*a*^	Median value (range)		
	Children >1 mo old	Neonates	Total
No. of patients	39	18	57
Mean no. of observations/patient	7.6	5.3	6.9
Sex (male:female)	21:18	12:6	33:24
Wt (kg)	14.8 (3–62.2)	2.04 (0.69–5.08)	7.5 (0.69–62.2)
Ht (cm)^*b*^	97.9 (45–170)	48 (36–52)	72 (36–170)
Age (yr)	3.3 (0.12–15.8)	0.05 (0.01–0.19)	0.88 (0.01–15.82)
PMA (wk)	NA^*c*^	37 (26–44)	NA
PNA (days)	1,204.5 (43.8–5,774.3)	17 (4–69)	321.2 (4–5,774.3)
eGFR (ml/min/1.73 m^2^)	78.94 (6.43–160.3)	42.8 (5.4–95.2)	62.06 (5.4–160.3)
Serum creatinine concn (μmol/liter)	41 (27–308)	44.5 (21–265)	41 (21–308)

a PMA, postmenstrual age; PNA, postnatal age; eGFR, estimated glomerular filtration rate.

b Data on height were provided for 30 children above the age of 1 month. For the rest of these children and all the neonates, the median value for height (or length) corresponding to each patient's gender and age, obtained from the UK pediatric growth charts, was used.

c NA, not applicable.

### Population PK of teicoplanin in neonates and children.

**FIG 1:**
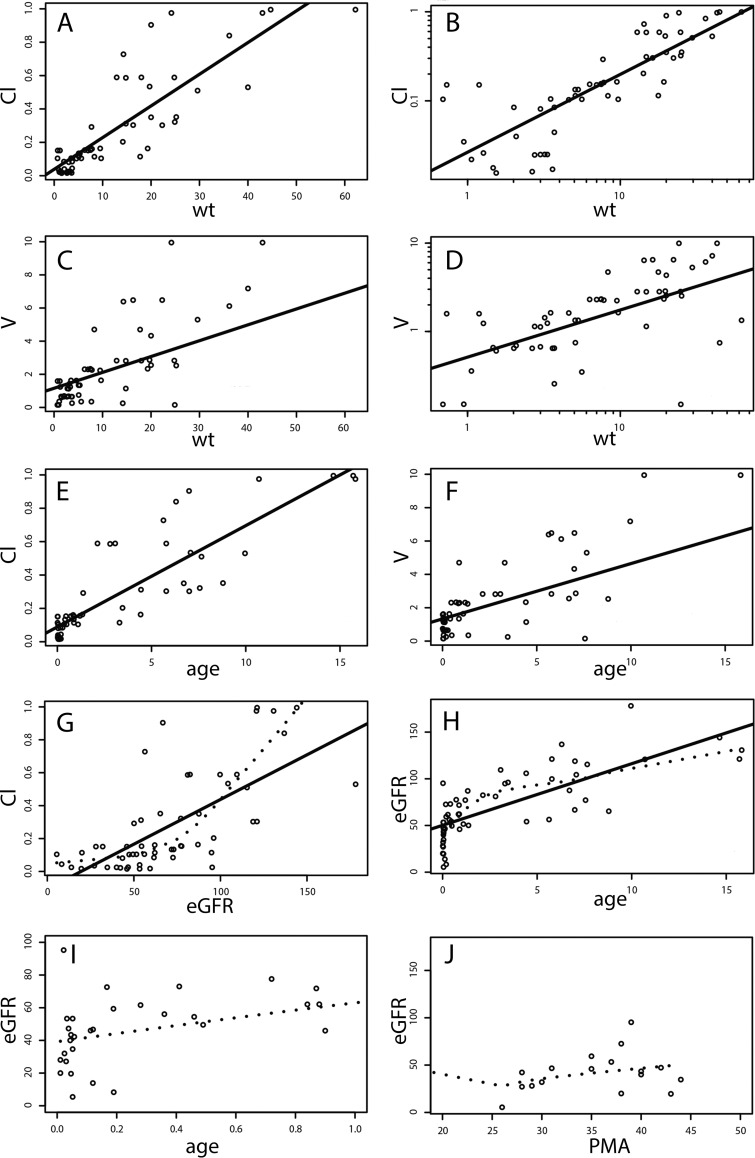
Relationships between covariates and the Bayesian posterior estimates for clearance (Cl) and volume (*V*) obtained from the base model. (A and B) Linear (A) and log_10_-log_10_ (B) relationships between Cl and weight (wt). The continuous line shows the linear regression. (C and D) Linear (C) and log_10_-log_10_ (D) relationships between volume and weight. (E and F) Linear relationships between clearance and age (in years) (E) and volume and age (in years) (F). (G and H) Relationships between clearance and eGFR (G) and eGFR and age (in years) (H). (I) The plotting of eGFR against age for infants <12 months old shows two distinct periods of change in eGFR with age (i.e., <0.25 and ≥0.25 years). The dashed line shows the LOWESS (locally weighted scatter plot smoothing), or local regression, which highlights a clearance-versus-eGFR relationship compatible with a power function, where GFR is the independent variable raised to a constant (pw) in panel G. (J) The relationship between eGFR and PMA (in weeks) is less informative than age (in years) for young infants.

Figure 1 shows the relationships between the posterior median estimates of the PK parameters for each patient from the base model and potentially relevant covariates. Relationships were apparent between both clearance (Cl) (Fig. 1A and B) and volume (Fig. 1C and D) and weight. Linear relationships between clearance and age (Fig. 1E) and volume and age (Fig. 1F) were also apparent. There was an exponential relationship between eGFR and clearance (Fig. 1G), with progressively higher estimates of clearance as eGFR increased. There were two distinct periods of change in eGFR as a function of age, which formed the basis for using separate functions that described the effect of changes in renal function on the elimination of teicoplanin (Fig. 1H and I). For neonates and young infants <3 months old, the postnatal age (PNA)/serum creatinine concentration ratio was used as a measure of renal function. For infants >3 months old and children, eGFR^pw^ was used (where pw is a constant). There was no relationship between serum albumin levels and the Bayesian estimates of clearance and volume; thus, albumin was not included as a covariate in the final model.

A comparison between the standard model without covariates and the final model is shown in Table 2. A number of candidate models that examined the impact of age on the weight-standardized elimination rate constant (Ke0) and weight-standardized volume using both linear scaling functions and sigmoidal functions were developed but did not describe the data better than allometric scaling using weight as a measure of size.

**TABLE 2:** Model comparison and model diagnostics with and without covariates for models

Parameter^*a*^	Value for:	
	Standard model (without covariates)	Final model (allometric without age)
No. of variables	4	7
Log-likelihood value	−1,262	−1,180
AIC	2,533	2,376
Pop/post bias (mg/liter)	3.8/−0.02	1.1/−0.15
Pop/post imprecision mg^2^/liter^2^	72.2/1.5	12.3/0.9
Pop *r*^2^	0.12	0.9
Post *r*^2^	0.8	0.92

a AIC, Akaike information criterion; Pop/post bias, population and posterior mean weighted error, respectively; Pop/post imprecision, population and posterior mean bias-adjusted weighted squared error, respectively; Pop *r*^2^ and Post *r*^2^, coefficient of determination for the linear regression of the observed-versus-predicted plots for the population and posterior fits, respectively.

**FIG 2:**
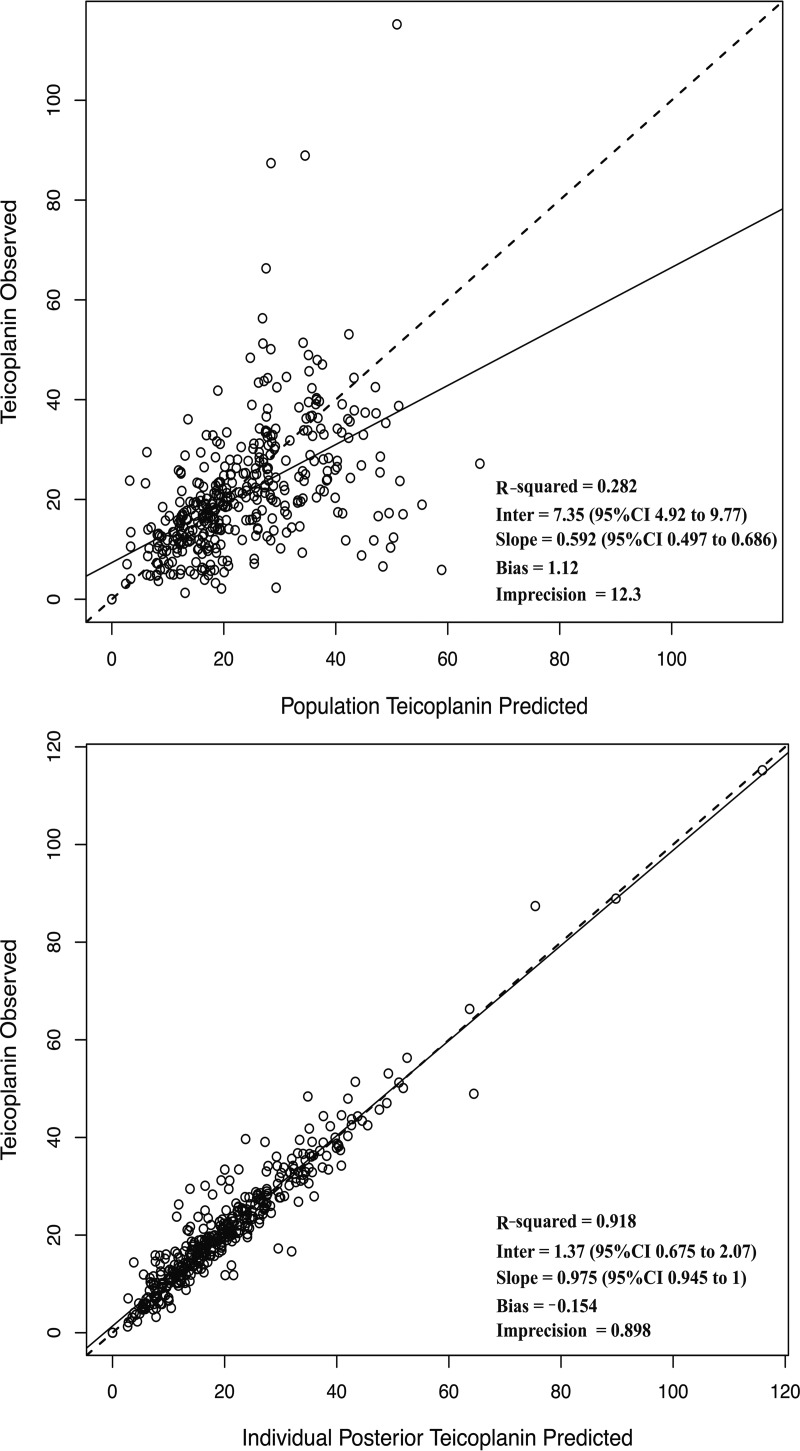
Scatter plots of observed-versus-predicted concentrations (in milligrams per liter) for the final PK model before (population) (top) and after (individual posteriors) (bottom) the Bayesian step using the median parameter values.

**FIG 3:**
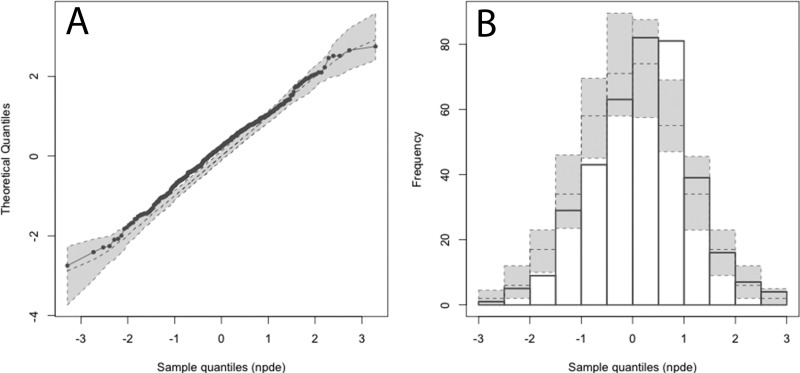
Normalized distribution predicted error (NPDE). (A) Q-Q plot of the distribution of the NPDE versus the theoretical normal [N (0, 1)] distribution. (B) Histogram of the distribution of the NPDE with the density of the standard Gaussian distribution overlaid. The results suggest an acceptable fit of the final model to the data.

**FIG 4:**
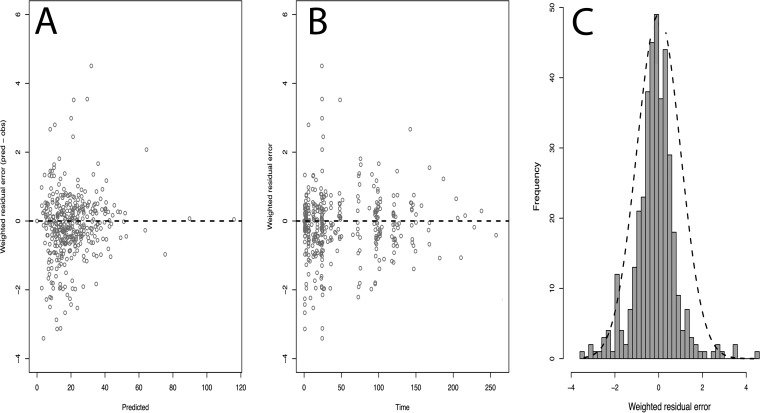
Weighted residual error (predicted − observed) distributions. (A) Weighted residuals versus predictions; (B) weighted residuals versus time; (C) histogram of residuals with a superimposed normal curve.

The parameter values (means, medians, and standard deviations) for the final model are summarized in Table 3. For the final model, the linear regression of observed versus Bayesian-predicted values had a coefficient of determination (*r*^2^) of 0.92, with measures of bias and precision of −0.15 mg/liter and 0.9 mg^2^/liter^2^, respectively (Table 2). The population and individual observed-versus-predicted plots of the final model are shown in Fig. 2. Normalized distribution prediction error (NPDE) results (Q-Q plot and histogram) are summarized graphically in Fig. 3. The weighted residual error distributions are shown in Fig. 4. Both the NDPE and the weighted residual error distributions suggest that the fit of the model to the data was acceptable.

**TABLE 3:** Population PK parameter estimates from the final model

Value	Parameter^*a*^						
	Ke0 (h^−1^)		V0 (liters)		Kcp (h^−1^)	Kpc (h^−1^)	pw
	Ke01	Ke02	V01	V02			
Mean	0.038	0.036	22.636	22.472	0.490	0.214	0.125
Median	0.03	0.025	19.52	22.7	0.23	0.12	0.07
SD	0.04	0.03	14.23	7.95	0.48	0.27	0.15

a Ke0, weight-standardized elimination rate constant. The elimination rate constant (Ke) = Ke0 · (wt/70)^−0.25^ · Renal (where Renal is the PNA/creatinine ratio if the patient is <0.25 year old and as eGFR^pw^ otherwise); V0, weight-standardized volume of the central compartment. Volume = V0 · (wt/70); Kcp and Kpc, first-order intercompartmental rate constants; pw, power function. The suffixes 1 and 2 denote the occasion, which referred to samples collected <96 h and ≥96 h, respectively, after treatment initiation.

### Performance and simulations to demonstrate the clinical utility of the teicoplanin dose optimization software.

The dose optimization software predicted the PK profiles of the individual patients and achieved a target with minimal bias and imprecision. Table 4 shows the bias, percentage of bias, imprecision, percentage of imprecision, and the coefficient of determination (*r*^2^) of the linear regression of the observed-versus-predicted concentrations for an infant and an older child. Individual weighted mean PK parameter values were obtained. The median (range) AUC from 0 to 24 h (AUC_0–24_) for each patient is shown.

**TABLE 4:** Summary of the individual prediction diagnostics from the dosing optimization software for the two patients^*a*^

Parameter^*b*^	Value for:	
	Patient 1 (infant)	Patient 2 (child)
*r*^2^	1	0.97
Bias	−0.42	0.4
% bias	−2.54	8.46
Imprecision	0.3	1.64
% imprecision	0.05	1.54
Median (range) 24-h AUC (mg · h/liter) along the treatment course	493.8 (355.8–574)	368.1 (318.9–388.4)

a Real data from the past were used in order to obtain a target of 15 mg/liter from day 3 of therapy.

b *r*^2^, coefficient of determination of the linear regression of the observed-versus-predicted concentrations; bias, mean weighted predicted-observed error; % bias, 100 × (mean weighted predicted-observed error/observation); imprecision, bias-adjusted mean squared error; % imprecision, 100 × (bias-adjusted mean squared error/observation).

**FIG 5:**
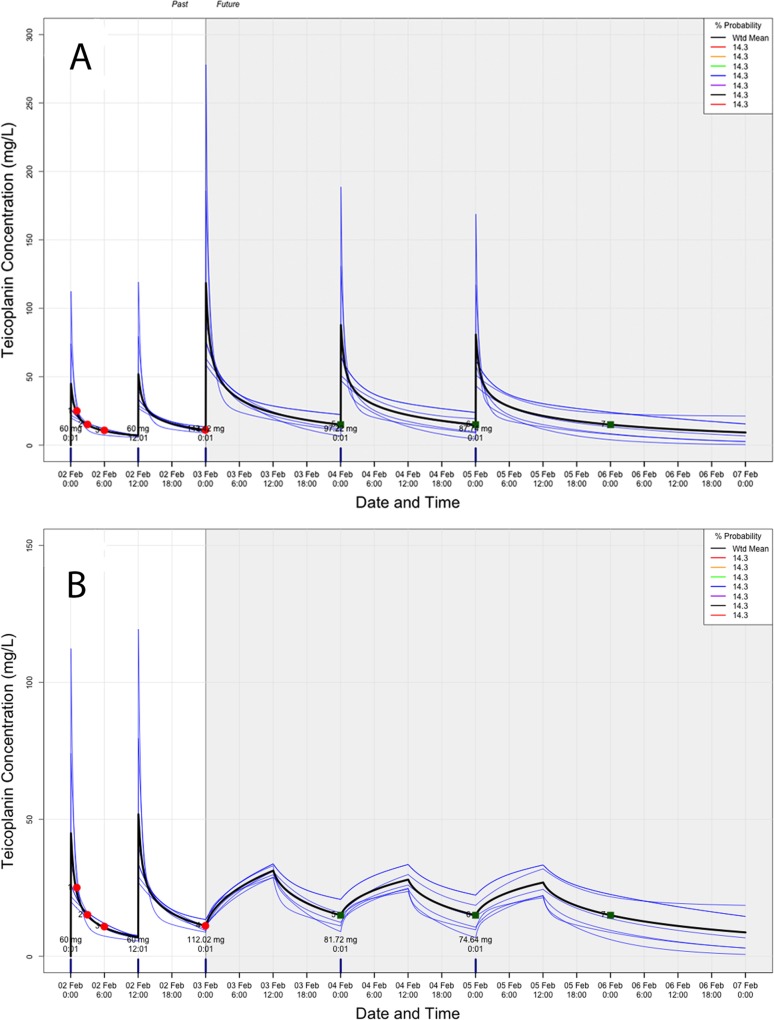
Representative plots from an infant patient receiving teicoplanin for a predose target of 15 mg/liter from day 3 of therapy following bolus administration (A) or a 12-h infusion (B). Red circles, measured concentrations; green squares, target concentration. (A) Bolus administration. Dose required on day 2, 143.4 mg (22.3 mg/kg); on day 3, 97.22 mg (15.4 mg/kg); on day 4, 87.74 (13.9 mg/kg). (B) Twelve-hour infusion. Dose required on day 2, 112.02 mg (17.8 mg/kg); on day 3, 81.72 mg (12.97 mg/kg); on day 4, 74.64 mg (11.8 mg/kg).

**FIG 6:**
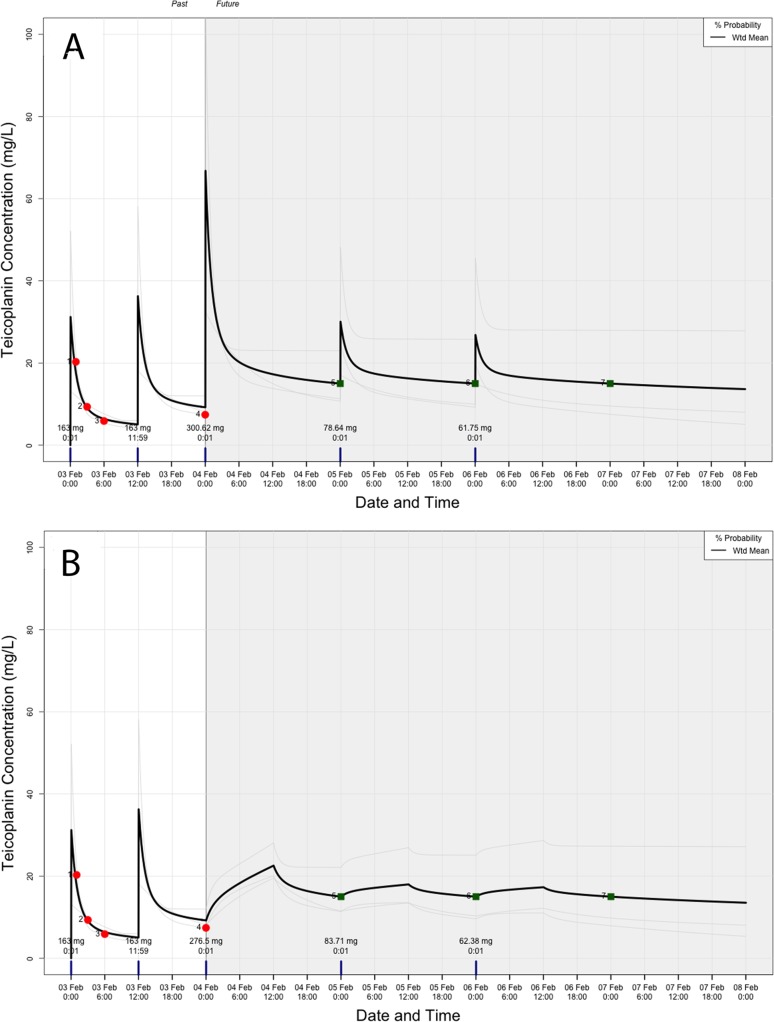
Representative plots from an older child receiving teicoplanin for a predose target of 15 mg/liter from day 3 of therapy following bolus administration (A) or a 12-h infusion (B). Red circles, measured concentrations; green squares, target concentration. (A) Bolus administration. Dose required on day 2, 300.62 mg (18.4 mg/kg); on day 3, 78.64 mg (4.8 mg/kg); on day 4, 61.75 mg (3.8 mg/kg). (B) Twelve-hour infusion. Dose required on day 2, 276.5 mg (17 mg/kg); on day 3, 83.71 mg (5.1 mg/kg); on day 4, 62.38 mg (3.8 mg/kg).

Figures 5 and 6 show representative plots from the dose controller for the infant and the older child, respectively. They represent the software-predicted PK profiles with their respective measured concentrations (circles) and the target concentration (squares). In the case of the infant, all optimized doses on the first two days of therapy to achieve a day 3 predose target of 15 mg/liter were 1.8 to 2.2 times higher than the currently recommended maintenance dose (10 mg/kg of body weight) for 12-h extended infusions and a 24-hourly bolus administration, respectively (Fig. 5). The regimen with daily 24-h infusions required slightly smaller dosages (Fig. 5B). In the case of the 5-year-old child, a higher bolus administration of 18.4 mg/kg (as opposed to the 10-mg/kg maintenance dose) was required to achieve the day 3 predose 15-mg/liter target, although this produced peaks of >60 mg/liter (Fig. 6A). For this patient, extended 12- and 24-h infusions, and even a multiple fractionated 12-hourly dose, would have been safer alternatives (Fig. 6B). Increased predicted and optimal dosages ranged from 13.9 mg/kg (1.4 times higher than the recommended maintenance dose) to 3.7, 2.6, and 2.2 mg/kg with the multiple fractionated doses to achieve a predose target of 15 mg/liter. There was no significant impact on dosages when the simulated patients had an average age-related eGFR higher than actual estimates. In these particular cases, they both required slightly increased dosages (≤2%).

The Monte Carlo simulations suggested that the best-performing regimen (3 loading doses of 25 mg/kg followed by a daily maintenance dose of 10 mg/kg) achieved *C*_min_ values of 15 to 60 mg/liter for only 30% of patients, and 13% had potentially toxic levels (>60 mg/liter).

## DISCUSSION

An improved understanding of the PK-pharmacodynamics (PD) of teicoplanin is fundamental to the optimal use of this agent (16). In this study, we developed a population PK model in neonates, infants, and older children to identify sources of PK variability and its explanatory covariates. The final pharmacokinetic model accounts for only 28% of the observed PK variability (Fig. 2, top), which is consistent with other population PK studies of children and adults (7, 9). The high and largely unexplained variability is a strong argument for the use of TDM to minimize both suboptimal and potentially toxic antimicrobial exposures (1, 2). The combination of a pharmacokinetic model with measurements from an individual patient and software-guided dosage adjustment provides a way in which future target concentrations can be achieved in a timely and optimally precise manner.

For teicoplanin, no relationship between the plasma drug concentration and toxicity has been established (10, 17–19), a situation that may reduce the incentive for clinicians to routinely monitor the drug. However, exposure control to maximize efficacy should not be neglected. The British National Formulary for Children (BNFC) suggests a therapeutic window of >15 to 60 mg/liter for children and adults (3). These recommendations are largely based on retrospective studies of MRSA infection (12, 13, 17, 20). Low drug exposures increase the probability of clinical failure and potentially promote the development of drug resistance (16, 21). This is the predominant argument for the routine monitoring of teicoplanin concentrations and active dosage adjustment.

Teicoplanin dosages that are adjusted by weight and/or renal function improve the achievement of target concentrations in adults (8, 22). However, high and unexplained PK variability makes this approach less effective in children and neonates. Hence, an alternative dosing strategy is required. Bayesian tools offer a way to achieve target concentrations in a timely and optimally precise manner (23). A number of Bayesian forecasting tools can be used to deliver dosage adjustment. In this study, the multiple-model algorithm embedded in the BestDose software package was used.

The following steps are required to achieve dosage individualization using the multiple-model algorithm. First, the entire patient population is described by a matrix of support points, which consist of individual sets of parameter values. Each support point has an associated probability, which reflects how well it describes individual patients within the population. The population is described by multiple support points, because there are both multiple patients and, typically, considerable interindividual pharmacokinetic variability. The set of support points constitutes the Bayesian prior probability distribution and is a mathematical summary of drug behavior in the population. In the next step, pharmacokinetic measurements are obtained from the patient whose dosage needs to be individualized. The probabilities of support points that best describe the PK of that patient are then revised. Those points that poorly describe the observed pharmacokinetics have their probability revised downward. Other points that perform better have their probability increased. Hence, each individual patient has the same set of support points as the total population, but with a revised probability distribution. In the final step, the dosage required to achieve the target concentration for the individual patient is calculated. This is achieved by calculating the dose that is required for each support point to achieve the desired target concentration. These dosages are then weighted by the probability of each support point and are summed to obtain a mean weighted dose.

The approach used in this study has several attractive features. First, dosage individualization can commence immediately, without waiting for steady state. Second, patients can be controlled without an explicit understanding of the sources or causes of pharmacokinetic variability. The control of critically ill patients who are clinically unstable (24, 25) represents an ongoing challenge. One limitation of current approaches (including this study) is that a patient's PK are assumed to be invariant. This can lead to poor fitting if the PK change and ultimately leads to suboptimal control. The only way to circumvent this problem at present is to use the most recent PK data to estimate a patient's PK and updated covariate information. In this case, the probability for each support point describing the PK for the new episode is recalculated, and these probabilities are then used to control subsequent dosing. The incorporation of the interacting multiple-model approach into dosage adjustment algorithms potentially provides a way to control unstable patients, but this has yet to be done (23).

Another interesting aspect of our work was the use of postnatal age (PNA) divided by the serum creatinine concentration (SCr) as a novel marker of renal function. In modeling and controlling drug behavior, it is not necessary to describe renal function using traditional equations that estimate GFR, although we did use the Schwartz equation for infants >3 months old. The goal in pharmacometrics is to find the best descriptors of drug behavior, which, in this case, are related to renal function and maturation. We believe that the PNA/SCr ratio has advantages over any other estimation of renal function, including the Schwartz equation, in that it does not use length (height), which can be notoriously inaccurate in infants. We have previously found the PNA/SCr ratio to be a useful predictor for both vancomycin (26) and gentamicin (27) PK in infants.

In conclusion, we present a tool to rapidly and accurately predict teicoplanin concentrations and thus to calculate doses that optimally achieve the desired concentrations in pediatrics. We further validate the PNA/SCr ratio as a novel predictor of renal drug elimination in neonates and young infants. A number of prospective clinical studies can now be considered. At the simplest level, these may consist of studies that have drug exposure as the primary endpoint. More-complex studies that have clinical outcomes and/or toxicity as primary endpoints will require a significantly larger number of patients and a multicenter design.

## MATERIALS AND METHODS

### Pharmacokinetic study of teicoplanin in children and neonates.

Pharmacokinetic data from 57 children (39 children aged 1 month to 16 years old and 18 neonates with a postmenstrual age [PMA] between 26 and 44 weeks) were available for model building. Patients were prospectively enrolled from two different hospitals in Liverpool (Alder Hey NHS Children's Foundation Trust and Liverpool Women's Hospital) over a 21-month period (April 2013 and January 2015). These PK data have been reported previously in two separate population models (1, 2). In this study, we combined these data sets to develop a joint population PK model fitted to data from neonates and older children that can be used for dosage individualization. Such an approach avoids the problem of having multiple pharmacokinetic models for the same drug, each with an arbitrarily chosen cutoff value for age or size.

The study was approved by the Medicines and Healthcare Products Regulatory Agency (clinical trial authorization no. 21362/00003/001-0001) and the National Research Ethics Service and Regional Committee (13/NW/0023). The trial was registered with the European Clinical Trials Database Registry under EudraCT no. 2012-005738-12. Written informed consent was obtained from parents and/or legal guardians.

Neonates with a PMA of ≤44 weeks received a loading dose of 16 mg/kg, followed by 8 mg/kg once daily via a 30-min intravenous (i.v.) infusion. Children >1 month old received three loading dosages of 10 mg/kg every 12 h, followed by 10 mg/kg once daily via an i.v. bolus infusion (2 to 5 min), according to the dosages currently recommended by the SPC (11). Plasma samples were collected during the first dosage interval and then at steady state (1, 3, 6, and 24 h postdose) on days 3 to 7 of therapy. Neonates weighing <1,000 g contributed two samples per dosing interval because of constraints on sample volume. The duration of the treatment course was at the discretion of the treating physician. All patients received teicoplanin for proven or suspected methicillin-resistant staphylococcal sepsis (due to either coagulase-negative staphylococci [CoNS] or MRSA) and/or central-line-associated bloodstream infection. Demographic variables included weight, height, age in years, PMA in weeks, postnatal age (PNA) in days, serum albumin concentration (in grams per liter), and serum creatinine concentration. The estimated glomerular filtration rate (eGFR) (Schwartz-Haycock) (28) was also available for each patient.

Teicoplanin concentrations were measured using a commercially available fluorescence polarization immunoassay (FPIA; Thermo Fisher Scientific, Germany). The limit of quantification (LOQ) was <3.0 mg/liter. The dynamic range was 3 to 100 mg/liter, and overall precision (intra- and interday variability) was <6%.

### Development of a population PK model.

A PK model was fitted to the data using Pmetrics, version 1.4.2 for R, statistical package 3.2.2, which utilizes the nonparametric adaptive grid (NPAG) algorithm (29). The inverse of the estimated assay variance was used to weight the data. Initially, a standard two-compartment model with time-delimited zero-order i.v. input and first-order elimination from the central compartment was developed. The standard model is described by differential equations 1a and 1b below:

(1a) $$\frac{dX\left(1\right)}{dt}=R(1)-(\text{Kcp}+\text{Cl}/V)\cdot X(1)+\left[\text{Kpc}\cdot X\left(2\right)\right]$$

(1b) $$\frac{dX\left(2\right)}{dt}=\text{Kcp}\cdot X(1)-\text{Kpc}\cdot X\left(2\right)$$

where *X*(1) and *X*(2) represent the amounts of teicoplanin (in milligrams) in the central (c) and peripheral (p) compartments, respectively; *R*(1) is the rate of infusion of the drug into the central compartment (in milligrams per hour); clearance (Cl) from the central compartment is measured in liters per hour; volume (*V*) is measured in liters; and the central and peripheral compartments are connected by the first-order rate constants Kcp and Kpc (measured per hour).

Once the standard model was developed, the potential effects of growth (size) and development (maturity) on the PK of teicoplanin were investigated. This investigation was conducted using clinical measures that are readily accessible, such as weight and age. We also examined the relationships between other potentially relevant covariates (e.g., serum albumin concentration) and the PK. The Bayesian individual posterior median estimates for clearance and volume of distribution were obtained from the standard model for each patient. These were then plotted against the covariates of interest in order to interrogate any possible relationships.

In neonates and children, clearance generally scales with size in a nonlinear manner that is best described using a power function. A scaling exponent of 0.75 is most frequently used (i.e., clearance is proportional to weight^0.75^). In addition, physiological maturation may also affect clearance, and this is especially important for neonates and young infants (30, 31). In the model-building process, we switched from using clearance to using Ke (measured per hour) as the first-order elimination rate constant, where Ke is calculated as Cl/*V* and is proportional to weight^−0.25^. While it is possible to estimate the scaling exponents directly, we chose to fix these values as previously described by others (24, 30, 31). There is a theoretical basis for using fixed scaling exponents that is related to fundamental relationships between size and a range of biological functions. The parameters Ke0 and V0 are weight-standardized parameters for the elimination rate constant and volume, respectively (31, 32). Since size was standardized to a 70-kg adult, Ke0 and V0 approximate adult values.

The potential impact of development (or maturation) on teicoplanin elimination was studied by using age (in years) for older children and PMA (in weeks) and/or PNA (in days) for neonates and younger infants. The effect of age (in years) was explored linearly with Ke and by using a sigmoidal maturation factor driven by PMA, as described previously (24). Ultimately, none of these functions were incorporated into the final model.

Finally, we also explored the impact of renal function on the PK of teicoplanin. Before doing this, we inspected the relationship between renal function and age in all patients to ensure that appropriate estimates of renal function were used in the model-building process. GFR was estimated (eGFR) using the Haycock-Schwartz formula (*k* · height/serum creatinine concentration), where *k* is the proportionality constant (33). A *k* value of 0.33, 0.45, and 0.41 was used for preterm neonates, term neonates, and older children, respectively. The different values of *k* reflect the smaller percentage of muscle mass in preterm infants than in term infants and older children (28, 34). If height (or length) was not directly recorded, values from UK pediatric growth charts for age and gender were used. We also considered the use of the PNA/SCr ratio as an alternative measure to eGFR for estimating renal function in relation to age in neonates and young infants.

The final structural model took the form defined by equations 2a and 2b:

(2a) $$\frac{dX\left(1\right)}{dt}=R(1)-\{\text{Kcp}+[\text{Ke}0\cdot {\left(\frac{\text{wt}}{70}\right)}^{-0.25}]\cdot \text{Renal}\}\cdot X(1)+\left[\text{Kpc}\cdot X\left(2\right)\right]$$

(2b) $$\frac{dX\left(2\right)}{dt}=\text{Kcp}\cdot X(1)-\text{Kpc}\cdot X\left(2\right)$$

with the output equation *Y*(1) = *X*(1)/*V*, which describes the time course of teicoplanin concentrations.

In these equations, Ke is calculated as [Ke0 · (wt/70)^(−0.25)^] · Renal [the exponent is −0.25 because Ke0 · (wt/70)^−0.25^ is algebraically equivalent to (Cl/*V*) · (wt/70)^0.75^]; *V* is calculated as V0 · (wt/70); and wt is the patient′s weight (in kilograms). A cutoff age in the maturation of renal function was apparent when eGFR was plotted against age (Fig. 1H and I) with an inflection point at 3 months (0.25 year). Hence, renal function (“Renal”) for infants and children >3 months old was described as the estimated glomerular filtration rate (eGFR) (measured in milliliters per minute per 1.73 m^2^) to an estimated power function (pw). If the patient's age was <0.25 year (i.e., <3 months), “Renal” was the PNA (in years) divided by the serum creatinine concentration (in micromoles per liter). Ke0 and V0 are the weight-standardized parameters for the elimination rate constant and volume, respectively.

There were two sampling periods, distinguished by an interoccasion variability (IOV) of 1 or 2 for concentrations collected at <96 h or ≥96 h, respectively. For the first sampling period (i.e., IOV = 1), Ke0 was designated Ke01 and V0 was designated V01. For the second sampling period (i.e., IOV = 2), Ke0 was designated Ke02 and V0 was designated V02.

The fit of each exploratory model to the data was assessed using a combination of the following: (i) the log-likelihood value, (ii) the Akaike information criterion (AIC), (iii) the coefficients of determination (*r*^2^) from the linear regression of the observed-predicted plots before and after the Bayesian step, (iv) minimization of bias and imprecisions of the observed-predicted plots, (v) the NPDE, and (vi) the distribution of the weighted residual errors. A model comparison was made using the above-named diagnostics in order to choose the best final model.

### Building the teicoplanin dose optimization software.

We incorporated the final population PK model into a teicoplanin multiple-model Bayesian adaptive dosing controller (the software “cartridge”). The controller is based on the concepts and software (BestDose) developed by the University of Southern California Laboratory for Applied Pharmacokinetics and Bioinformatics (LAPKB) (http://www.lapk.org) (35, 36). The teicoplanin cartridge included the final structural model equations relating input (dosing information) to output (concentrations of the drug in plasma) and the discrete joint probability distribution of the values of the equation variables (PK parameters) in the population, consisting of a discrete number of support points and their associated probabilities (the Bayesian prior). The cartridge was implemented in BestDose, version 0.2.4 for R, which used the cartridge and each patient's weight, age, PNA, serum creatinine level, eGFR, and teicoplanin dosing-concentration data to find the least biased and most precise dosage regimen relative to a target concentration, as described previously (35).

### Simulations to demonstrate the utility of the dose optimization software.

To demonstrate the potential value of the dose optimization software as a clinical tool, the software was used to predict the required dosage by day 2 of therapy to achieve a predetermined teicoplanin concentration (15 mg/liter) from day 3 in two representative subjects selected from the study population on the basis of age: (i) a critically ill infant (age, 5 months [0.46 year]; weight, 6.3 kg; eGFR, 63.84 ml/min/1.73 m^2^) and (ii) a critically ill older child (age, 5.78 years; weight, 16.3 kg; eGFR, 108.41 ml/min/1.73 m^2^). We used the real past concentration-time and dosing data from these two patients during the first dosing interval plus a 48-h trough (*n*, 4 observations) to predict the optimized dose and infusion time for achieving the desired target concentration safely (i.e., avoiding peaks of >60 mg/liter, regarded as potentially toxic levels [3]). A “past” data file contained the observed concentrations for each patient. A “future” data file, containing the required timings of future dosages and the target, an initial guess of the likely future dose(s) that would be required, and the infusion time, was prepared. The same patients were also investigated with different simulated age-related average eGFRs (77 and 127 ml/min/1.73 m^2^, respectively) in order to evaluate the impact of renal function on the patient's PK profile (37).

The dose optimization software was tested by comparing the estimated predicted PK profile plot against the observations, as well as by the linear regression of the observed-versus-predicted concentrations for each individual patient. From the predicted concentrations based on the median individual Bayesian posterior parameter distribution, we calculated the bias, which is equal to the mean weighted predicted error (∑ wpe/*N*), where wpe is (predicted concentration − actual concentration)/(SD for each prediction/observation), *N* is the number of observations, and the percentage of bias. We also computed the imprecision, which is the mean bias-adjusted weighted squared error (∑ wspe/*N*-mwpe^2^), and its respective percentage for each patient and each experimental run. The weighted mean individual PK parameter values and an average 24-h AUC, estimated by trapezoidal approximation to hourly predictions for each subject, were also computed by the software.

Monte Carlo simulations were performed to assess the proportion of patients receiving fixed regimens with a trough concentration (*C*_min_) of 15 to 60 mg/liter by day 3 of therapy and the proportion with potentially toxic concentrations (>60 mg/liter). Four candidate regimens were examined: (i) 3 loading dosages consisting of 30 mg/kg every 12 h (q12h), followed by 20 mg/kg q24h; (ii) 3 loading dosages consisting of 20 mg/kg q12h, followed by 15 mg/kg q24 h; (iii) 3 loading dosages consisting of 25 mg/kg q12 h, followed by 10 mg/kg q24 h; and (iv) 2 loading dosages consisting of 30 mg/kg q12 h, followed by 10 mg/kg q24 h. One hundred concentration-time profiles were simulated for each regimen. The range of the covariates used in the simulations (i.e., weight, age, and the renal function descriptor) was the same as for the original population. The simulations were performed using Pmetrics.
